# Comparative Efficacy of the Air-Q Intubating Laryngeal Airway during General Anesthesia in Pediatric Patients: A Systematic Review and Meta-Analysis

**DOI:** 10.1155/2016/6406391

**Published:** 2016-06-23

**Authors:** Eun Jin Ahn, Geun Joo Choi, Hyun Kang, Chong Wha Baek, Yong Hun Jung, Young Cheol Woo, Si Ra Bang

**Affiliations:** ^1^Department of Anesthesiology and Pain Medicine, Inje University Seoul Paik Hospital, Seoul, Republic of Korea; ^2^Department of Anesthesiology and Pain Medicine, Chung-Ang University College of Medicine, Seoul 156-755, Republic of Korea

## Abstract

Air-Q® (air-Q) is a supraglottic airway device which can be used as a guidance of intubation in pediatric as well as in adult patients. We evaluated the efficacy and safety of air-Q compared to other airway devices during general anesthesia in pediatric patients by conducting a systematic review and meta-analysis. A total of 10 studies including 789 patients were included in the final analysis. Compared with other supraglottic airway devices, air-Q showed no evidence for a difference in leakage pressure and insertion time. The ease of insertion was significantly lower than other supraglottic airway devices. The success rate of intubation was significantly lower than other airway devices. However, fiberoptic view was better through the air-Q than other supraglottic airway devices. Therefore, air-Q could be a safe substitute for other airway devices and may provide better fiberoptic bronchoscopic view.

## 1. Introduction

There are many supraglottic airway devices (SADs) which are used for the management of a difficult airway and as a conduit for tracheal intubation [1]. The endotracheal intubation assisted by SADs has many advantages including easy insertion, better alignment of the glottis opening, and continuous patient oxygenation and ventilation. Moreover, the haemodynamic stress response to intubation by SAD is less than that of direct laryngoscope [2]. Also, these devices can be a good alternative for patients with previous history of difficult intubation, restricted neck mobility, and stability of cervical spine [3]. Moreover, SAD provides the ability to overcome upper airway obstruction and provision of a hands-free airway with a relatively straightforward path to the larynx [4].

Among these SADs, Air-Q Intubating Laryngeal Airway (air-Q, Mercury Medical, Clearwater, FL, USA) is a SAD intended for allowing for airway maintenance under general anesthesia as well as an aid for tracheal intubation with a cuffed tracheal tube in both adults and pediatric patients. The design of air-Q includes a large airway tube inner diameter (ID), a short airway tube length, and a tethered, removable standard 15 mm circuit adapter [5]. Also, specially designed ridges inside mask cavity provide lateral stability to help prevent tip from bending backward and improve mask seal. These features enable direct insertion of a curved shaft, the lack of a grill in the ventilating orifice, and an easily removable airway adapter [5].

Several studies reported the efficacy of air-Q in pediatric patients comparing other airway devices such as laryngeal mask airway (LMA), i-gel, Aura-I, Cobra Perilaryngeal Airway (CobraPLA), and fiberoptic guided intubation. However, the findings are variable and the reported outcomes from several studies are conflicting. To date, no systematic review nor meta-analysis regarding air-Q has been performed. Therefore, we aimed to evaluate the efficacy of air-Q compared with other airway devices in pediatric patients.

## 2. Methods

This systematic review was conducted following the guidelines of the PRISMA statement [6].

### 2.1. Systematic Search

We conducted a systematic review and meta-analysis for RCTs which compared the air-Q with the other airway device during general anesthesia in pediatric patients. Literature searches were conducted in MEDLINE, EMBASE, Cochrane Library, KoreaMed, KMBASE, and Google Scholar inclusive at June 1, 2014, and updated at September 2015. The search strategy combining free text and related search is attached in the Appendix.

### 2.2. Selection of Included Studies

The study's inclusion and exclusion criteria were determined before systematic search. Two review authors (Eun Jin Ahn and Si Ra Bang) independently scanned the titles and abstracts identified by the variety of search strategies described above. If the report was determined not eligible from the title or abstract, the full paper was retrieved. Potentially relevant studies, chosen by at least 1 author, were retrieved and evaluated in full-text versions. The articles that met the inclusion criteria were assessed separately by 2 authors (Eun Jin Ahn and Si Ra Bang), and any discrepancies were resolved through discussion. If agreement would not be reached the dispute was resolved with the help of third investigator (Hyun Kang).

### 2.3. Inclusion and Exclusion Criteria

We included randomized controlled trials which compared the air-Q and the other airway device during general anesthesia in pediatric anesthesia. The group used air-Q as an airway device considered as an experimental group. Otherwise, group used other laryngeal mask airway devices and fiberoptic bronchoscope as an airway device considered as a control group. We excluded data from abstracts, posters, case reports, comments or letters to the editor, reviews, and animal studies. There was no limitation in language to select studies.

### 2.4. Study Outcomes

The outcome data were divided into two series as if the air-Q is used as a SAD or used as a conduit of tracheal intubation. In series of air-Q used as SAD, the outcomes included success rate of insertion of airway device, oropharyngeal leakage pressure, insertion time, and ease of insertion airway device. In series of air-Q used as a conduit of intubation, the outcomes included success rate of intubation, the number of attempts, intubation time, and fiberoptic glottis view. Also, complications including desaturation, sore throat, blood staining of device, and laryngospasm were evaluated.

We compared the number of attempts through the rate of successful intubation at first attempt. Ease of insertion of airway device or intubation through airway device was analyzed by comparing the number of easiest level of difficulty. The extracted outcome data of fiberoptic glottis view grade were divided into 4 grades [13–16] (visible vocal cords/visible vocal cord with posterior epiglottis/visible vocal cord with anterior epiglottis/no visible vocal cord) or 5 grades [8–12] (visible vocal cords/visible vocal cord with posterior epiglottis/vocal cord anterior visible <50% obstruction/visible vocal cord with anterior epiglottis, >50% obstruction/no visible vocal cord). The scenarios were divided into best and worst which include the incidence of fiberoptic glottis view grade with visible vocal cord and no visible vocal cord.

We performed subgroup analyses for comparing groups, SADs, and fiberoptic guided intubation. SADs were also divided into Aura-I, CobraPLA, i-gel, and LMA series which include LMA-Unique and LMA-Flexible. We also performed subgroup analyses based on the generations of SADs. First-generation devices included LMA-Unique, LMA-Flexible, and CobraPLA. The second-generation devices included Aura-I, i-gel, and LMA-Fastrach. The sensitivity analysis was performed to rule out the excessive effect of single study on heterogeneity.

### 2.5. Validity Scoring

The quality of eligible studies was assessed independently by two authors (Chong Wha Baek and Yong Hun Jung) of our review group using the tool of “risk of bias” according to Review Manager software (version 5.3, the Cochrane Collaboration, Oxford, UK). The quality was evaluated based on the following seven potential sources of bias: random sequence generation; allocation concealment; blinding of the participants and their parents; blinding of outcome assessment; incomplete outcome data; selective reporting. The methodology of each trial was graded as “high,” “low,” or “unclear,” to reflect a high risk of bias, low risk of bias, and uncertainty of bias, respectively (Table 3).

### 2.6. Data Extraction

All interrelated data in each included study were independently extracted onto a spreadsheet by 2 authors (Eun Jin Ahn and Geun Joo Choi) and cross-checked. The spreadsheet included the following indexes: (1) name of first author, (2) year, (3) name of journal, (4) study design, (5) registration of clinical trial, (6) risk of bias, (7) number of patients in study, (8) sex of patients, (9) age, (10) weight, (11) height, (12) ASA physical status, (13) type of surgery, (14) number and experience of device user, (15) number of allowance of insertion trials, (16) device size, (17) induction method, (18) maintenance agent, (19) use of muscle relaxant, and (20) intervention/control. If there were missing data, we made attempts to contact the authors to chase the data of included studies. And if the author could not be contacted, the data were acquired from estimated value in the figure or graph.

### 2.7. Statistical Analysis

We conducted this meta-analysis by Review Manager (version 5.3, the Cochrane Collaboration, Oxford, UK) and Comprehensive Meta-Analysis software (version 2.0, Biostat, Englewood, NJ, USA). Three authors (Eun Jin Ahn, Geun Joo Choi, and Young Cheol Woo) independently input all data to the software. For dichotomous data, we calculated pooled risk ratio (RR), odds ratio (OR), and 95% confidence intervals (CIs). If the 95% CI included a value of 1, we considered the difference not to be statistically significant. We calculated the mean difference for continuous data, also reported with 95% CI. We used the Chi-squared test and the *I*-squared test for heterogeneity. A level of 10% significance (*P* < 0.1) for the Chi-squared statistic or *I* ^2^ greater than 50% was considered to indicate considerable heterogeneity. The Mantel–Haenszel random-effect model was used for these studies. The Mantel–Haenszel fixed model was used for studies that did not demonstrate significant heterogeneity [17, 18]. For data expressed with median and interquartile range, we changed mean and standard deviation via data extraction method from Cochrane handbook for systematic reviews of intervention [17].

We estimated publication bias using Begg's funnel plot and Egger's linear regression test. If the funnel plot was visually asymmetrical or the *P* value was found to be <0.1 using Egger's linear regression test, the presence of a possible publication bias was identified [19].

## 3. Result

**Figure 1:**
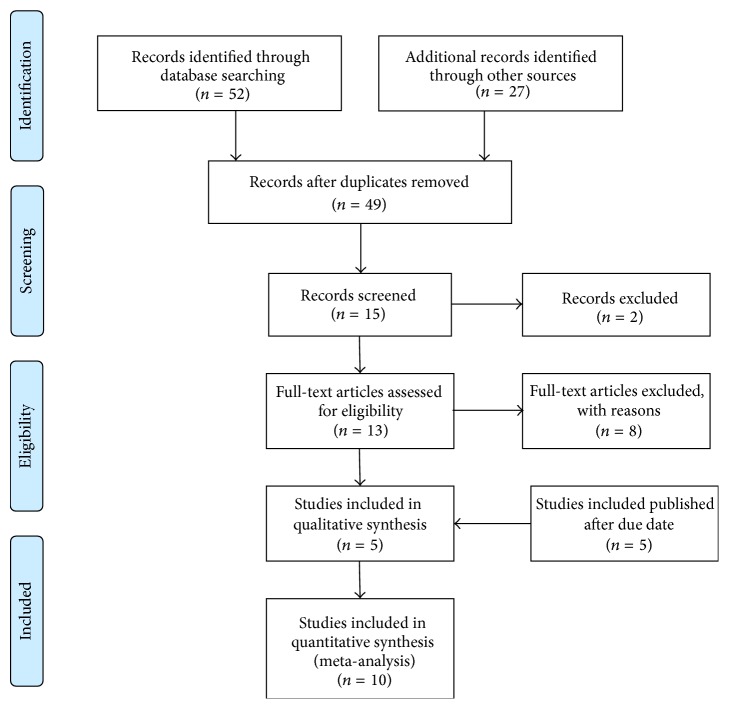
PRISMA flow diagram of the search, inclusion, and exclusion of randomized controlled trials.

The search of MEDLINE, EMBASE, Cochrane Library, KoreaMed, KMBASE, and Google Scholar produced 79 studies. After adjusting for duplicates, 49 studies remained. Of these, 34 studies were discharged because it appeared that these studies were out of interest after reviewing the title and abstracts. The full texts of the remaining 15 studies were reviewed in more detail and 10 studies were excluded [2, 5, 20–27]. Also, by searching studies which were published after first search date (June 1, 2014), 5 more studies were found [11, 12, 14–16]. Thus, 10 studies with a total of 789 patients were included in the final analysis, met the inclusion criteria, and were included in this systematic review and meta-analysis [7–16] (Figure 1).

Two of these studies used self-pressurizing air-Q rather than original air-Q which require cuff inflation [10, 12]. The air-Q was compared with three types of airway managements: other supraglottic airway devices (SADs) [7–16] and fiberoptic tracheal intubation [7]. SAD types include LMA-Flexible in one RCT [14]; LMA-Unique in two RCTs [8, 10]; Aura-I in three RCTs [9, 15, 16]; Cobra Perilaryngeal Airway in one RCT [13]; and i-gel in two RCTs [11, 12].

Among SADs comparing air-Q, LMA-Flexible, LMA-Unique, and Cobra Perilaryngeal Airway were first-generation SADs [8, 10, 13, 14]. Aura-I and i-gel were second-generation SADs [9, 11, 12, 15, 16]. The summary of included studies is described through Tables 1 [Table tab2]–3.

### 3.1. Risk of Bias

In all the included studies, the random sequence generation method was performed and seven studies used allocation concealment [8, 10, 12–16]. Three RCTs were registered in clinical trial [8, 9, 12, 16] and no incomplete data was reported. All included studies reported no sponsorship. The overall risks of bias are shown in Table 4.

### 3.2. Air-Q Used as SAD

#### 3.2.1. Oropharyngeal Leakage Pressure

The oropharyngeal leakage pressure (OLP) was compared to other SADs in nine studies [8–16]. The combined results showed no evidence for a difference, MD −0.00 (−2.19 to 2.18), *P* < 0.00001, *I* ^2^ = 91%. Performing subgroup analysis based on the generations of the airway devices, the OLP in air-Q showed no evidence of difference compared to first generations of SADs [8, 10, 13, 14], RR −0.04 (95% CI −3.51 to 3.42), *P* < 0.00001, *I* ^2^ = 91%. Also, the OLP in air-Q comparing to second generations of SADs showed no evidence of difference [9, 11, 12, 15, 16], RR −0.04 (95% CI −3.24 to 3.33), *P* < 0.00001, *I* ^2^ = 93%. However, by combining three studies [9, 15, 16] among second generations of SADs which compared OLP of air-Q and Aura-I, the OLP was significantly higher in Air-Q group than Aura-I with substantial heterogeneity, MD 2.09 (0.47 to 3.71), *P* = 0.13, *I* ^2^ = 51%.

#### 3.2.2. Success Rate of Device Insertion

Success rate of device insertion was compared in seven studies [8–10, 12, 14–16]. The combined results showed no evidence for a difference, RR 1.00 (0.98 to 1.03), *P* = 0.90, *I* ^2^ = 0%. In most studies, the success rate of device insertion was 100%. Only in one study [16], the success rate of device insertion of air-Q was 97.5% (39/40).

#### 3.2.3. Device Insertion Time

Device insertion time was compared in eight studies [8–11, 13–16]. The combined results showed no evidence for a difference, MD 0.69 (−1.00 to 2.38), *P* < 0.00001, *I* ^2^ = 91%. However, in subgroup analysis by combining three studies [9, 15, 16] which compared device insertion time of air-Q and Aura-I, the insertion time appeared significantly longer in air-Q group than Aura-I with substantial heterogeneity, MD 3.57 (0.97 to 6.17), *P* = 0.0002, *I* ^2^ = 88%. Throughout sensitivity analysis excluding the study of Kleine-Brueggeney et al. [16], the result became significant without heterogeneity which implies that the insertion time of air-Q was longer than Aura-I, MD 1.90 (1.06 to 2.73), *P* = 0.42, *I* ^2^ = 0%. Insertion time of air-Q was compared to LMA series in three studies [8, 10, 14]. The combined results showed no evidence for a difference in insertion time of air-Q compared to LMA series, MD −0.48 (95% CI −2.29 to 1.32), *P* = 0.001, *I* ^2^ = 85%. However, in single study [11], insertion time of air-Q compared to i-gel was significantly shorter (16.7 [4.1] versus 19.6 [4.7]).

#### 3.2.4. Ease of Insertion

**Figure 2:**
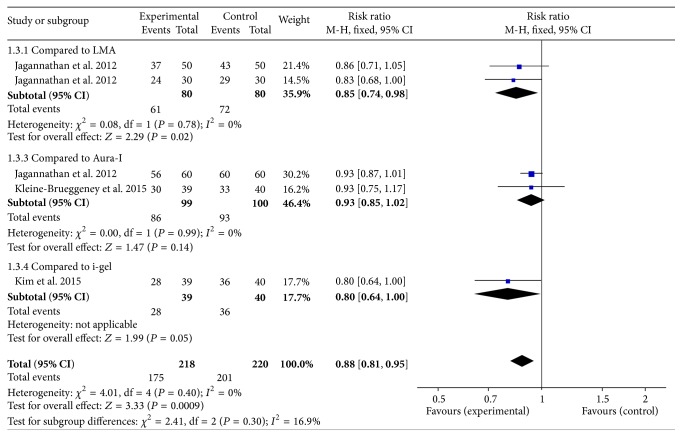
Forest plot for ease of insertion. The figure depicted individual trials as filled squares with relative size of sample size and solid line as the 95% confidence interval of the difference. The diamond shape indicates the pooled estimate and uncertainty for the combined effect.

Ease of insertion was compared to other SADs in five studies [8–10, 12, 16]. The combined results showed the ease of insertion in air-Q was significantly lower than other SADs, RR 0.88 (95% CI 0.81 to 0.95), *P* = 0.40, *I* ^2^ = 0% (Figure 2).

### 3.3. Air-Q Used as a Conduit of Intubation

#### 3.3.1. Total Success Rate of Intubation

The total success rate of intubation was compared to other airway devices in five studies [7, 9, 11, 13, 16]. Among these studies, four studies performed the assisted fiberoptic intubation through the airway devices. However, in the single study of Kleine-Brueggeney et al. [16], both groups performed blind intubation through the airway devices rather than assisted fiberoptic intubation. The combined results of five studies could not reveal the significant outcome (RR 0.97, 95% CI 0.85 to 1.11, *P* = 0.09, *I* ^2^ = 51%). Performing sensitivity analysis with removing Kleine-Brueggeney et al.'s study, the result showed a tendency of the total success rate of air-Q which was lower than other airway devices, RR 0.95 (95% CI 0.88 to 1.04), *P* = 0.22, *I* ^2^ = 31%. In the single study of Kleine-Brueggeney et al. [16], the total success rate was 15% (6/40) with the air-Q and 3% (1/40) with the Aura-I.

#### 3.3.2. The Rate of Successful Intubation at First Attempt

The rate of successful intubation at first attempt in air-Q was compared to other airway devices in four studies [7, 9, 11, 13]. The combined results showed no evidence of difference in the rate of successful intubation at first attempt in air-Q compared to other airway devices. However, the result showed a tendency of the rate of successful intubation at first attempt in air-Q which was higher than other airway devices, RR 1.08 (95% CI 0.99 to 1.17), *P* = 0.29, *I* ^2^ = 20%.

#### 3.3.3. Time to Intubate

The time to intubation was compared to other airway devices in five studies [7, 9, 11, 13, 16]. The combined results showed no evidence of a difference in the time to intubation, MD 1.341 (95% CI −4.44 to 7.13), *P* = 0.04, *I* ^2^ = 60%. Subgroup analyses were performed based on airway devices, SADs, fiberoptic guided. The combined results of four studies [9, 11, 13, 16] which compared air-Q and other SADs showed no evidence of difference, MD −0.18 (95% CI −6.20 to 5.84), *P* = 0.07, *I* ^2^ = 58%. Also, in a single study of Sohn et al. [7], there was no difference in time to intubation between free-handed and air-Q assisted tracheal intubation (*P* = 0.13).

### 3.4. Fiberoptic View Score

#### 3.4.1. Best Scenario

**Figure 3:**
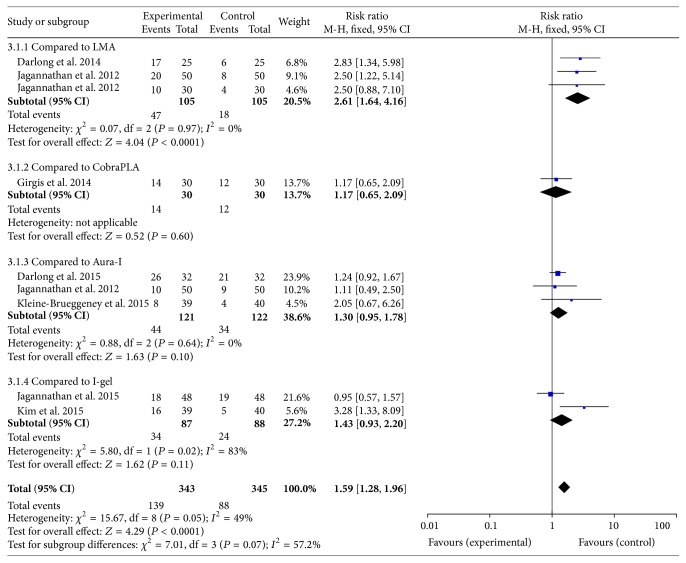
Forest plot for the fiberoptic view of best scenario. The figure depicted individual trials as filled squares with relative size of sample size and solid line as the 95% confidence interval of the difference. The diamond shape indicates the pooled estimate and uncertainty for the combined effect.

The fiberoptic view of best scenario in air-Q was compared to other SADs in nine studies [8–16]. The best scenario of fiberoptic view was better through the air-Q than other SADs, RR 1.59 (95% CI 1.28 to 1.96), *P* = 0.05, *I* ^2^ = 49% (Figure 3). Performing subgroup analysis based on the generations of the airway devices, the best scenario of fiberoptic view in air-Q was better than first generations of SADs [8, 10, 13, 14], RR 2.03 (95% CI 1.42 to 2.92), *P* = 0.19, *I* ^2^ = 37%. Moreover, the best scenario of fiberoptic view in air-Q was better than second generations of SADs [9, 11, 12, 15, 16], RR 1.35 (95% CI 1.05 to 1.75), *P* = 0.15, *I* ^2^ = 40%.

#### 3.4.2. Worst Scenario

The fiberoptic view of worst scenario in air-Q was compared to other SADs in nine studies [8–16]. The worst scenario of fiberoptic view showed no evidence of difference in the air-Q compared to other SADs, RR 1.10 (95% CI 0.59 to 2.07), *P* = 0.54, *I* ^2^ = 0%. Performing subgroup analysis based on the generations of airway devices, the worst scenario of fiberoptic view in air-Q showed no evidence of difference compared to first generation of SADs, RR 0.78 (95% CI 0.30 to 2.03), *P* = 0.57, *I* ^2^ = 0%. Also, there was no evidence of difference in the worst scenario of fiberoptic view in air-Q compared to second generation of SADs, RR 1.44 (95% CI 0.62 to 3.34), *P* = 0.25, *I* ^2^ = 27%.

### 3.5. Safety Analyses

#### 3.5.1. Blood Staining on Device

The incidence of blood staining on device was compared in six studies [8–10, 12–14]. There was no evidence of difference for the incidence of blood staining on device, RR 0.57 (95% CI 0.22 to 1.50), *P* = 0.53, *I* ^2^ = 0%. However, in the single study of Girgis et al. [13], the incidence of blood staining on device was significantly higher in patients with CobraPLA than Air-Q (30% versus 6.7%).

#### 3.5.2. Sore Throat

The incidence of sore throat was compared in three studies [10, 13, 14]. The air-Q showed lower incidence of sore throat than other airway devices, RR 0.45 (95% CI 0.24 to 0.85), *P* = 0.41, *I* ^2^ = 0%.

#### 3.5.3. Desaturation

The incidence of desaturation was compared in five studies [8, 9, 12–14]. There was no evidence of difference for the incidence of desaturation, RR 1.70 (95% CI 0.30 to 9.59), *P* = 0.79, *I* ^2^ = 0%.

#### 3.5.4. Laryngospasm

The incidence of laryngospasm was compared in three studies [8, 13, 14]. There was no evidence of difference for the incidence of laryngospasm, RR 0.73 (95% CI 0.15 to 3.58), *P* = 0.78, *I* ^2^ = 0%.

#### 3.5.5. Publication Bias

There was no evidence of publication bias detected by Egger's linear regression test and funnel plot. There was no *P* value of Egger's regression test that was showed to be <0.1 which is indicative of publication bias.

## 4. Discussion

The major finding of our meta-analysis was that air-Q is more difficult to insert than other airway devices. Also, the total success rate of intubation and the rate of successful intubation at first attempt showed a tendency lower than other airway devices. However, the fiberoptic view in best scenario was better in air-Q than other SADs. Also, in a safety analysis, the incidence of sore throat was lower in air-Q than other airway devices. The combined analysis of OLP, success rate of device insertion, insertion time, time to intubation, and the worst scenario of fiberoptic view could not reveal the difference between air-Q and other airway devices.

A tendency of higher success rate of intubation at first attempt in air-Q than other airway devices might be caused by better view of fiberoptic videoscope of air-Q. Using fiberoptic bronchoscope to guide tracheal intubation through a SAD is an established technique for securing the airway in children when conventional laryngoscopy is failed [11]. Also, SAD provides the ability to overcome upper airway obstruction and provision of a hands-free airway with a relatively straightforward path to the larynx [4, 7, 11]. The total success rate of blind intubation in Kleine-Brueggeney et al.'s study was only 10% (6/60), while the total success rate of air-Q involving four studies which used air-Q as a fiberoptic guidance for intubation [7, 9, 11, 13] was 82.5% (147/178). Because of high incidence of worst scenario in pediatric patients and potential injury to the epiglottis or the glottis, blind intubations through SADs are not recommended in several studies [16, 28, 29]. The infants have large and floppy epiglottis preventing visualization of glottis [29]. In a pilot study of Sinha, the incidence of best scenario of fiberoptic bronchoscopy was higher in infants compared to other studies aimed at children [28–30]. In conclusion, it is recommended that SGA be used as a fiberoptic bronchoscope guidance of tracheal intubation not as a guide of blind intubation in children. Also, further studies should be needed to apply the result of this study according to patients' age.

Performing the subgroup analysis, OLP was higher in air-Q than in Aura-I. The possible reasons for better OLP in air-Q are due to the unique features which include (i) curved and rigid airway tube which approximates the upper oropharyngeal airway with the glottis, (ii) mask ridges which improve the transverse stability of the bowl and support the lateral cuff seal, and (iii) raised mask heel [8, 10, 25]. Even though i-gel has shown higher airway leak pressure compared with other airway devices in children [31], the subgroup analysis showed no evidence of difference. These unique features of air-Q, especially raised mask heel, would have caused better OLP; otherwise they caused the following issues. The insertion of air-Q was more difficult and needed longer time compared to other SADs.

There are SADs which are simply “airway device” which may or may not protect against aspiration in the event of regurgitation. These SADs are called first generation including classic LMA, LMA-Flexible, laryngeal tube, and Cobra Perilaryngeal Airway. Second-generation SADs including LMA ProSeal, LMA Supreme, and i-gel provide a higher leak pressure and offer a drain tube to separate the respiratory and gastrointestinal tracts and minimize the risk of aspiration [32, 33]. Air-Q, classified in second-generation SAD [34], showed no evidence of difference in OLP compared to first-generation SADs in this meta-analysis. This result suggests that air-Q has little advantage in OLP compared to first-generation SADs and no further studies would be needed.

Through safety analysis, the incidence of sore throat was lower in patients in air-Q than other airway devices including CobraPLA and LMA. This result can be associated with the lower incidence of blood staining on device in air-Q than CobraPLA. Comparing CobraPLA and air-Q, “Cobra head” of the CobraPLA is more stiff in comparison with the cuff of other SADs including the air-Q which lead to more mucosal injury [13]. Therefore, we suggest that air-Q might be less traumatic SAD compared to other airway devices.

In two studies, self-pressurized air-Q was used rather than air-Q with balloon [10, 12]. Self-pressurized air-Q has an inner aperture at the junction of the airway tube and the mask cuff, creating an open airspace between the two and allowing the pressure to be self-regulated, which might provide easier device insertion and reduced risk for prolonged overinflation of the cuff and pressure-related injuries to the pharyngeal mucosa without the need for cuff pressure monitoring [10]. To rule out the effect of difference between air-Q which requires cuff inflation and self-pressurized air-Q, subgroup and sensitive analysis was performed. However, no result was changed through subgroup analysis and sensitive analysis.

There are some limitations in our study. First, because of air-Q being a newly developed SAD and a large variety of study designs, the number of studies involved in each subgroup was small. Second, large difference in the operator's experience (from trainee to senior anesthesiologists) could be a confounding factor.

In summary, we found that the air-Q could be a safe substitute for other intubating laryngeal mask airway devices and might provide better fiberoptic bronchoscopic view and shorter time to guide intubation.

## Figures and Tables

**Table 1:** Summary of studies included.

Source	Number of patients	Sex (M/F)	Age	Weight	Height	ASA
Sohn et al. 2014 [7]	80	Not reported	8 (6)^*∗*^	8 (2)	71 (8)	1, 2, 3
Jagannathan et al. 2012 [8]	100	Not reported	19 (6)^*∗*^	11 (1)	Not reported	1, 2
Jagannathan et al. 2012 [9]	120	85/35	2 (1)^*∗*^	12 (4)	89 (23)	1, 2, 3
Jagannathan et al. 2012 [10]	60	38/22	7 (2)^*∗*^	25 (3)	Not reported	1, 2, 3
Jagannathan et al. 2015 [11]	96	Not reported	2.2	12.3	84.5	1, 2, 3
Kim et al. 2015 [12]	79	76/3	2.8 (1.9)^*∗*^	14.6 [4.9]	88.6 [16.8]	1, 2
Girgis et al. 2014 [13]	60	30/20	3.9 (1.5)^*∗*^	16.5 [3.1]	91.8 [11.2]	1, 2
Darlong et al. 2014 [14]	50	39/11	8^*∗*^	6.5 (2.1)	7.2 (1.9)	1, 2
Darlong et al. 2015 [15]	64	45/19	8.7 (2.8)^*∗*^	6.6 (1.7)	Not reported	1, 2
Kleine-Brueggeney et al. 2015 [16]	80	43/37	4.3	16.3	116	1, 2, 3

ASA: American Society of Anesthesiology Classification; age: years or months^*∗*^; FOB: fiberoptic bronchoscopy.

**Table 2 tab2:** Summary of studies included.

Source	Amount and experience of device user	Type of surgery	Allowance of insertion trials	Intervention/control
Sohn et al. 2014 [7]	Attending or trainee, trainee having minimal prior experience with pediatric fiberoptic bronchoscopes and watched a video outlining the steps for fiberoptic guided tracheal intubation through an air-Q before participating	Elective surgical procedures requiring tracheal intubation under general anaesthesia	3	Air-Q/fiberoptic tracheal intubation

Jagannathan et al. 2012 [8]	Two anesthesiologists experienced in using both devices	Elective outpatient surgery in the supine position	2	Air-Q/LMA-Unique

Jagannathan et al. 2012 [9]	Five study investigators who used the air-Q for tracheal intubation in at least 50 patients and who have minimal experience with the Aura-I prior to this study	Elective surgery under general endotracheal anaesthesia	3	Air-Q/Aura-I

Jagannathan et al. 2012 [10]	Three study investigators experienced in the use of both devices	Elective outpatient surgery with planned airway management with a LMA device	2	Air-Q/LMA-Unique

Jagannathan et al. 2015 [11]	Anaesthesiology trainees, resident or fellow from clinical anaesthesia 2, 3, or 4 who had not previously performed FOB-guided tracheal intubation through an SGA in children → and received a brief lecture and viewed a video outlining the steps for FOB-guided tracheal intubation through an SGA	Elective surgery under general endotracheal anaesthesia	3	Air-Q/i-gel

Kim et al. 2015 [12]	Two anesthesiologists experienced in inserting supraglottic airway devices in at least 100 pediatric patients	Elective surgery under general endotracheal anaesthesia	2	Air-Q/i-gel

Girgis et al. 2014 [13]	Not reported	Elective surgery under general endotracheal anaesthesia	2	Air-Q/CobraPLA

Darlong et al. 2014 [14]	Not reported	Cataract or glaucoma surgery	3	Air-Q/Flexible laryngeal mask

Darlong et al. 2015 [15]	Not reported	Elective surgery with tracheal intubation	3	Air-Q/Aura-I

Kleine-Brueggeney et al. 2015 [16]	Senior anesthesiologists from the pediatric anaesthesia division	Elective ophthalmic surgery	3	Air-Q/Aura-I

**Table 3:** Summary of studies included.

Source	Device size	Use of muscle relaxant	Induction method	Maintenance agent
Sohn et al. 2014 [7]	According to manufacturer guidelines based on the patient's weight	Rocuronium	8% sevoflurane, 70% nitrous oxide, rocuronium 0.6 mg/kg	Sevoflurane

Jagannathan et al. 2012 [8]	Size 2 and size 1.5	No	Sevoflurane with 70% nitrous oxide and fentanyl 1 *μ*g/kg	Sevoflurane

Jagannathan et al. 2012 [9]	Based on the manufacturer guidelines	Rocuronium	Sevoflurane with 70% nitrous oxide and fentanyl 1 *μ*g/kg	Sevoflurane

Jagannathan et al. 2012 [10]	Air-Q: size 2 and LMA: 2.5	No	Sevoflurane with 70% nitrous oxide and fentanyl 1 *μ*g/kg	Sevoflurane with 60% nitrous oxide

Jagannathan et al. 2015 [11]	Based on manufacturer guidelines	Rocuronium	70% nitrous oxide and sevoflurane 8%	Sevoflurane 3%

Kim et al. 2015 [12]	Based on manufacturer guidelines	No	Propofol 2 mg/kg or 6% sevoflurane	3-4% sevoflurane

Girgis et al. 2014 [13]	Based on manufacturer guidelines	Atracurium	Premedication with midazolam 0.5 mg·kg, 8% sevoflurane, 100% oxygen, fentanyl 1 *μ*g/kg, atracurium 0.5 mg/kg	Not reported

Darlong et al. 2014 [14]	Depending upon body weight	Atracurium	Sevoflurane 2–8%, fentanyl 1 *μ*g·kg, atracurium 0.25 mg/kg	Isoflurane 1-2%

Darlong et al. 2015 [15]	Depending upon body weight	Atracurium	Sevoflurane 2–8%, fentanyl 1 *μ*g·kg, atracurium 0.25 mg/kg	Isoflurane 1-2%

Kleine-Brueggeney et al. 2015 [16]	Based on manufacturer guidelines	Atracurium	Sevoflurane 6% for inhalation induction, propofol 4 mg/kg or thiopental 6 mg/kg for intravenous induction, fentanyl or alfentanil, atracurium	Not commented

LMA: laryngeal mask airway; ETT: endotracheal tube.

**Table 4:** Risk of bias in included randomized controlled trials.

Biases/references	Random sequence generation	Allocation concealment	Incomplete outcome data	Blinding of participants	Blinding of outcome assessment	Selective reporting	Other bias
Sohn et al. 2014 [7]	Low risk	Unclear	Low risk	Unclear	Unclear	Low risk	Low risk
Jagannathan et al. 2012 [8]	Low risk	Low risk	Low risk	Unclear	Unclear	Low risk	Low risk
Jagannathan et al. 2012 [9]	Low risk	Unclear	Low risk	Unclear	Unclear	Low risk	Low risk
Jagannathan et al. 2012 [10]	Low risk	Low risk	Low risk	Unclear	Unclear	Low risk	Low risk
Jagannathan et al. 2015 [11]	Low risk	Unclear	Low risk	Unclear	Unclear	Low risk	Low risk
Kim et al. 2015 [12]	Low risk	Low risk	Low risk	Unclear	Unclear	Low risk	Low risk
Girgis et al. 2014 [13]	Low risk	Low risk	Low risk	Unclear	Unclear	Low risk	Low risk
Darlong et al. 2014 [14]	Low risk	Low risk	Low risk	Unclear	Unclear	Low risk	Low risk
Darlong et al. 2015 [15]	Low risk	Low risk	Low risk	Unclear	Unclear	Low risk	Low risk
Kleine-Brueggeney et al. 2015 [16]	Low risk	Low risk	Low risk	Unclear	Unclear	Low risk	Low risk

## Appendix

### A. Search Terms for MEDLINE

1. randomized controlled trial.pt
2. randomized controlled trial$.mp
3. controlled clinical trial.pt
4. controlled clinical trial$.mp
5. random allocation.mp
6. exp double-blind method/
7. double-blind.mp
8. exp single-blind method/
9. single-blind.mp
10. or/1–9
11. clinical trial.pt
12. clinical trial$.mp
13. exp clinical trial/
14. (clin$ adj25 trial$).mp
15. ((singl$ or doubl$ or tripl$ or trebl$) adj25 (blind$ or mask$)).mp
16. random$.mp
17. exp research design/
18. research design.mp
19. or/11–18
20. 10 or 19
21. Case report.tw.
22. Letter.pt.
23. Historical article.pt.
24. Review.pt.
25. or/21–24
26. 20 not 25
27. Air-Q.mp.
28. Air Q.mp.
29. air q.mp.
30. air-q.mp.
31. or/27–30
32. exp pediatrics/
33. exp child/
34. exp infant, Newborn/
35. child.mp
36. children.mp
37. pediatric.mp
38. infant.mp
39. neonate.mp
40. or/32–39
41. 31 and 40
42. 26 and 41

### B. Search Terms for EMBASE

1. randomized controlled trial$.mp.
2. “controlled clinical trial (topic)”/exp
3. controlled AND clinical AND trials
4. controlled clinical trial$.mp.
5. “randomization”/exp
6. “random allocation”/exp
7. random allocation.mp.
8. double-blind.mp.
9. single-blind.mp.
10. #1 OR #2 OR #3 OR #4 OR #5 OR #6 OR #7 OR #8 OR #9
11. “clinical trial (topic)”/exp
12. clinical AND trial$.mp.
13. random$.mp.
14. rct
15. #11 OR #12 OR #13 OR #14
16. #10 OR #15
17. “case study”/exp
18. “case report”/exp
19. “abstract report”/exp
20. “letter”/exp
21. #17 OR #18 OR #19 OR #20
22. #16 NOT #21
23. “air q”.mp
24. “air-q”.mp
25. Air-Q
26. Air q
27. #23 OR #24 OR #25 OR #26
28. “child”/exp
29. “newborn”/exp
30. “infant”/exp
31. child.mp
32. children.mp
33. pediatric.mp
34. infant.mp
35. neonate.mp
36. #28 OR #29 OR #30 OR #31 OR #32 OR #33 OR #34 OR #35
37. #27 and #36
38. #22 and #3
